# Effect of tidal volume on gastric insufflation during laparoscopic cholecystectomy: a strictly retrospective observational study

**DOI:** 10.3389/fsurg.2025.1708814

**Published:** 2026-01-16

**Authors:** Xiaolong Zhao, Jinyang Zhao, Chengjiang Zhang

**Affiliations:** Department of Anesthesiology, The First Affiliated Hospital of Xi’an Jiaotong University, Yulin Hospital, Yulin, Shanxi, China

**Keywords:** end-tidal carbon dioxide (PetCO_2_), end-tidal oxygen (ETO_2_), facemask ventilation, gastric insufflation, laparoscopic cholecystectomy, peak inspiratory pressure (PIP)

## Abstract

**Background:**

Laparoscopic cholecystectomy (LC) is the gold-standard minimally invasive gallbladder removal procedure. Optimal ventilation during LC requires positive end-expiratory pressure (PEEP) and low tidal volumes (TV) to prevent gastric insufflation (GI), which may cause regurgitation and cardiopulmonary complications.

**Method:**

This strictly retrospective observational study analyzed routine collected data from 60 patients undergoing laparoscopic cholecystectomy between January 2022 and December 2023. Patients were categorized into three groups based on anesthesia records of delivered tidal volumes (6, 8, or 10 mL/kg) during facemask ventilation. While group assignment was performed retrospectively, ventilation parameters were standardized per institutional protocol, ensuring consistent clinical delivery. Patients were divided into Group 1 (6 mL/kg), Group 2 (8 mL/kg), and Group 3 (10 mL/kg). Gastric insufflation was assessed via ultrasonography, and respiratory parameters end-tidal carbon dioxide (PetCO_2_), end-tidal oxygen (ETO_2_), and peak inspiratory pressure (PIP) were recorded.

**Result:**

GI incidence was significantly higher in Group 3 (60%) vs. Group 1 (15%, *p* = 0.0079) and Group 2 (20%, *p* = 0.0225). Group 3 showed greater antral area expansion post-ventilation (504.1 ± 109.8 mm^2^ vs. 420.1 ± 47.1 mm^2^, *p* = 0.001). PetCO_2_ and ETO_2_ levels differed significantly across groups (*p* < 0.001).

**Conclusion:**

The study reveals that Group 2’s facemask ventilation may improve preoxygenation and minimize gastric insufflation during laparoscopic cholecystectomy anesthesia induction. Further research is needed due to the small sample size, ultrasonography accuracy issues, and a single-center scenario.

## 1 Introduction

Laparoscopic cholecystectomy, first introduced in the late 1980s, has become the gold standard for gallbladder removal due to its advantages over open surgery, including reduced postoperative pain, shorter hospitalization, and faster recovery (1–3). However, the required pneumoperitoneum creates significant physiological alterations, particularly affecting respiratory mechanics and gas exchange (4–7). Carbon dioxide insufflation elevates intra-abdominal pressure (typically 11–17 mmHg), causing diaphragmatic displacement and impaired ventilation-perfusion matching that necessitate specialized ventilation strategies (7, 8).

These respiratory challenges have driven research into optimal ventilation approaches. Current evidence supports protective lung ventilation incorporating positive end-expiratory pressure (PEEP) and low tidal volumes to maintain oxygenation while preventing barotrauma (9, 10). However, clinicians must balance these benefits against potential hemodynamic consequences and surgical field requirements (11, 12). This balance becomes particularly critical when considering gastric insufflation—a common but understudied complication of improper ventilation that may lead to regurgitation, aspiration, and compromised surgical conditions (13, 14).

The anesthesiology literature has extensively compared ventilation modes pressure-controlled ventilation (PCV) vs. volume-controlled ventilation (VCV) (15, 16) and evaluated muscle relaxant effects (17), with some studies examining pressure thresholds for gastric insufflation (18, 19). However, despite tidal volume's direct clinical relevance, evidence guiding volume-specific ventilation during laparoscopic cholecystectomy remains surprisingly limited (20, 21). Current protocols often employ generic thresholds without pneumoperitoneum-specific adjustments (18, 22), potentially increasing gastric insufflation risks.

While previous studies have primarily focused on ventilation pressures (e.g., PCV vs. VCV) and their effects on gastric insufflation (15, 20, 21), the role of tidal volume remains underexplored despite its direct clinical applicability. Current guidelines lack evidence-based recommendations for volume-specific ventilation during laparoscopic cholecystectomy, often defaulting to generic thresholds (18, 22). Our study addresses this gap by systematically comparing three tidal volumes (6, 8, and 10 mL/kg), using routine clinical data collected from existing patient records to refine anesthesia protocols and mitigate gastric insufflation, a preventable yet understudied complication. By linking volume-specific outcomes to intraoperative metrics (e.g., antral area, PetCO_2_), we provide actionable insights for optimizing perioperative safety.

This investigation employs rigorous methodology to advance perioperative practice. Real-time ultrasonographic measurement of antral area provides objective quantification of gastric distension, overcoming limitations of previous qualitative assessments. Combined with continuous respiratory monitoring respiratory parameters end-tidal carbon dioxide (PetCO_2_), end-tidal oxygen (ETO_2_), and peak inspiratory pressure (PIP), we establish precise correlations between tidal volumes and clinical outcomes. Our three-group comparison (6 vs. 8 vs. 10 mL/kg) specifically targets the knowledge gap in volume selection during pneumoperitoneum, with data derived from existing clinical records.

The study's clinical implications are substantial. By establishing evidence-based tidal volume recommendations, we may reduce gastric insufflation incidence while maintaining optimal respiratory function. This could decrease perioperative complications ranging from surgical field obstruction to aspiration pneumonia. Furthermore, our ultrasonographic protocol offers clinicians a practical tool for intraoperative monitoring. These advancements may lead to updated ventilation guidelines specifically tailored to laparoscopic cholecystectomy's unique physiological demands.

## 2 Method

### 2.1 Research design

This strictly retrospective observational study analyzed routine clinical data from 60 patients who underwent elective laparoscopic cholecystectomy at The First Affiliated Hospital of Xi'an Jiaotong University, Yulin Hospital, between January 2022 and December 2023. All consecutive patients scheduled for elective laparoscopic cholecystectomy during the study period who met the predefined inclusion and exclusion criteria were included, and no patients were selected or excluded based on intraoperative or postoperative outcomes. Ventilation settings were determined by a standardized institutional protocol rather than individual clinician preference.

This retrospective analysis was selected to efficiently evaluate real-world clinical outcomes across varying ventilation volumes while minimizing patient recruitment bias. The data used were routine clinical records, which align with the study's goal of analyzing existing data to investigate the relationship between tidal volume and gastric insufflation. By leveraging existing anesthesia records and standardized surgical reports, we ensured consistent data collection while maintaining clinical relevance to routine practice.

This strictly retrospective observational study included 60 patients who underwent elective laparoscopic cholecystectomy between January 2022 and December 2023. Ventilation parameters (tidal volumes: 6, 8, or 10 mL/kg) were routinely documented in anesthesia records as part of routine care, and data were later analyzed by group assignment. While analyzing data retrospectively, all patients received care under our standardized ventilation protocol, with parameters automatically recorded by anesthesia machines. Since all consecutive eligible cases were included and ventilation management followed a standardized protocol, potential selection bias was minimized, although not fully eliminated due to the non-randomized design Group assignment was based on these objective recordings rather than clinician selection. The patient's age range was 29–84 years, and patients were assigned to three groups (*n* = 20 per group) according to their treatment as following: Group 1 (tidal volume = 6 mL/kg), Group 2 (tidal volume = 8 mL/kg), and Group 3 (tidal volume = 10 mL/kg), and were separated into GI+ and GI− subgroups based on the presence or absence of gastric insufflation in the 3 groups. The current study was approved by the Ethics Committee of our hospital (approval number YLH202412221). Written informed consents from all patients were obtained in any experimental work with humans.

### 2.2 Anesthesia induction

After the upper arm intravenous insertion, the operating room started electrocardiography, oxygen saturation, and noninvasive blood pressure monitoring. Midazolam, sufentanil citrate, propofol, and rocuronium worked intravenously to induce anesthesia. When the eyelid muscle response disappeared, an expert anesthesiologist implemented an oropharyngeal airway.

### 2.3 Ultrasonography

All ultrasounds were performed by one of three board-certified anesthesiologists with ≥2 years of gastric point-of-care ultrasound (POCUS) experience, who completed a standardized 4-h training module on antral measurement (including 20 supervised scans) prior to data collection. All examinations were performed with the patient in the supine position after induction of anesthesia and before tracheal intubation, and the same position and probe orientation were used for both the pre-ventilation and post-ventilation scans. A portable ultrasound locator with a 3.5 MHz probe scanned the sagittal plane beneath the xiphoid process before and 120 s during facemask ventilation post-anesthesia induction. The antrum, abdominal aorta sagittal plane, liver left lobe, and superior mesenteric artery were imaged. The cross-sectional antral area (*π*D1D2/4) was calculated by measuring the antrum's anteroposterior (D1) and superoinferior (D2) diameters three times for mean values in a frozen image by the same operator, and the mean value was used to reduce intra-observer variability and improve reproducibility. A comet-tail indication or antral hypertrophy indicated gastric insufflation. Ultrasonic measurements were repeated before intubation at the designated site. During surgery, sevoflurane 3% maintained anesthesia.

### 2.4 Intraoperative evaluation

The surgical team, blinded to the patient's assigned ventilation group, graded gastric distension (Grades 1–4) upon laparoscope insertion. This 4-point scale was adapted from standardized laparoscopic assessments of surgical field compromise (23, 24), with good inter-rater reliability (*κ* = 0.79) achieved during pre-study training using video simulations. Anesthesia providers recorded ventilation parameters separately to maintain blinding. The three-port laparoscopic cholecystectomy was performed after sterilizing and draping. When the laparoscope entered the abdomen, the surgeon assessed gastric distension using the following criteria: Grade 1 (gastric empty, lesser curvature not reaching gallbladder), Grade 2 (mild tension/slight fullness, lesser curvature contacting gallbladder), Grade 3 (moderate filling with high tension, lesser curvature covering gallbladder but manageable with retraction), or Grade 4 (severe distension requiring gastric tube placement). Grade definitions were informed by quantitative ultrasound correlations from prior validation studies (15, 20, 21). Pneumoperitoneum was maintained at 11–17 mmHg with carbon dioxide. Grades 1–2 allowed adequate exposure of the hepatobiliary triangle.

### 2.5 Respiratory parameters

Recorded oxygen saturation (30 s), real-time PIP (60 s), end-tidal carbon dioxide partial pressure (PetCO_2_) (90 s), and end-tidal oxygen concentration (ETO_2_) (120 s) during facemask inhalation.

### 2.6 Eligibility criteria

#### 2.6.1 Inclusion criteria

Patients undergoing elective laparoscopic cholecystectomy under general anesthesia were included if they were aged 29–84 years with ASA physical status I–II confirmed by two anesthesiologists and body mass index between 18.5 and 35 kg/m^2^. All participants adhered to preoperative fasting protocols (≥6 h for solids, ≥2 h for clear liquids) and had complete anesthesia machine recordings (tidal volume, PetCO_2_, PIP) paired with full ultrasonographic antral area measurements pre-/post-ventilation. Written informed consent was obtained for all enrolled patients treated at the study hospital during January 2022–December 2023.

#### 2.6.2 Exclusion criteria

Patients were excluded for history of GERD or hiatal hernia >2 cm, prior upper GI surgery (except uncomplicated appendectomy), or upper GI endoscopy within 72 h preoperatively. Additional exclusions comprised difficult airway (Mallampati score ≥3, thyromental distance <6 cm), active respiratory infection (within 2 weeks) or chronic lung disease (COPD GOLD ≥2), pregnancy, missing ventilation/ultrasound data, or intraoperative conversion to open cholecystectomy.

### 2.7 Retrospective group assignment and data validation

Group assignments were determined retrospectively by analyzing routine clinical anesthesia machine data (Datex-Ohmeda Aisys CS2, GE Healthcare), which documented actual delivered tidal volumes during each procedure. Patients were categorized into three groups based on the recorded ventilation parameters: Group 1 (6 mL/kg) included patients receiving 5.5–6.5 mL/kg, Group 2 (8 mL/kg) comprised those with 7.5–8.5 mL/kg, and Group 3 (10 mL/kg) contained cases with 9.5–10.5 mL/kg, allowing a ±0.5 mL/kg tolerance to account for minor clinical variations.

Inclusion required: (1) complete machine records of delivered tidal volumes, (2) protocol-compliant ventilation (±0.5 mL/kg of target), and (3) complete ultrasound series. Cases were excluded for missing data (*n* = 7) or major protocol violations (*n* = 2). Two independent anesthesiologists reviewed all anesthesia records to verify group assignments, with any discrepancies resolved through consensus discussion. Only procedures with complete ventilation data throughout the entire induction period were included in the analysis, and machine calibration logs were cross-checked to ensure measurement accuracy during the study period. This method of retrospective grouping-maintained alignment with actual clinical practice while ensuring physiological relevance of the compared tidal volume ranges.

### 2.8 Sample size considerations

While this exploratory study initially enrolled 60 patients (20/group) based on clinical feasibility, *post-hoc* power analysis revealed the sample provided 82% power (*α* = 0.05, two-tailed) to detect large effects (Cohen's *h* ≥ 0.8) in gastric insufflation incidence between groups, using chi-square tests. This effect magnitude aligns with prior studies showing 50%–65% risk differences in ventilation-related complications (14, 15).

### 2.9 Statistical analysis

All analyses were performed using SPSS 27 (IBM Corp.) and R 4.1.2. Continuous variables with normal distribution (confirmed by Shapiro–Wilk tests) are presented as mean ± standard deviation, while non-parametric data are shown as median [IQR]. Categorical variables are expressed as frequencies (percentages). To assess measurement consistency, 30 randomly selected patients underwent duplicate scans by two operators. Inter-observer Interclass correlation coefficients (ICC) were calculated for both antral diameters and area, confirming excellent reliability (ICC > 0.90 for all measures). To account for potential confounders, we performed logistic regression with gastric insufflation as the dependent variable and tidal volume group, age, BMI, and gender as covariates. Model fit was assessed using Hosmer-Lemeshow tests, and multicollinearity was evaluated via variance inflation factors (all VIF < 2). *Post-hoc* power for the regression model was 78% to detect an effect size of *f*^2^ = 0.25 at *α* = 0.05. Primary group comparisons utilized one-way ANOVA with Tukey *post-hoc* tests for continuous outcomes and chi-square/Fisher's exact tests for categorical outcomes. Multivariate linear regression was additionally performed to evaluate the independent association between tidal volume groups and gastric insufflation while adjusting for BMI, age, and gender, with model assumptions verified through *Q*-*Q* plots and residual analysis (VIF <5 for all covariates confirmed no concerning collinearity). Bivariate correlations (Pearson/Spearman) assessed relationships between respiratory parameters, and intraclass correlation coefficients (ICC) evaluated ultrasound measurement reliability. Effect sizes are reported as Cohen's *d* for ANOVA comparisons (0.2 = small, 0.5 = medium, 0.8 = large) and odds ratios with 95% CIs for regression models. All tests were two-tailed with statistical significance set at *p* < 0.05.

## 3 Result

**Figure 1:**
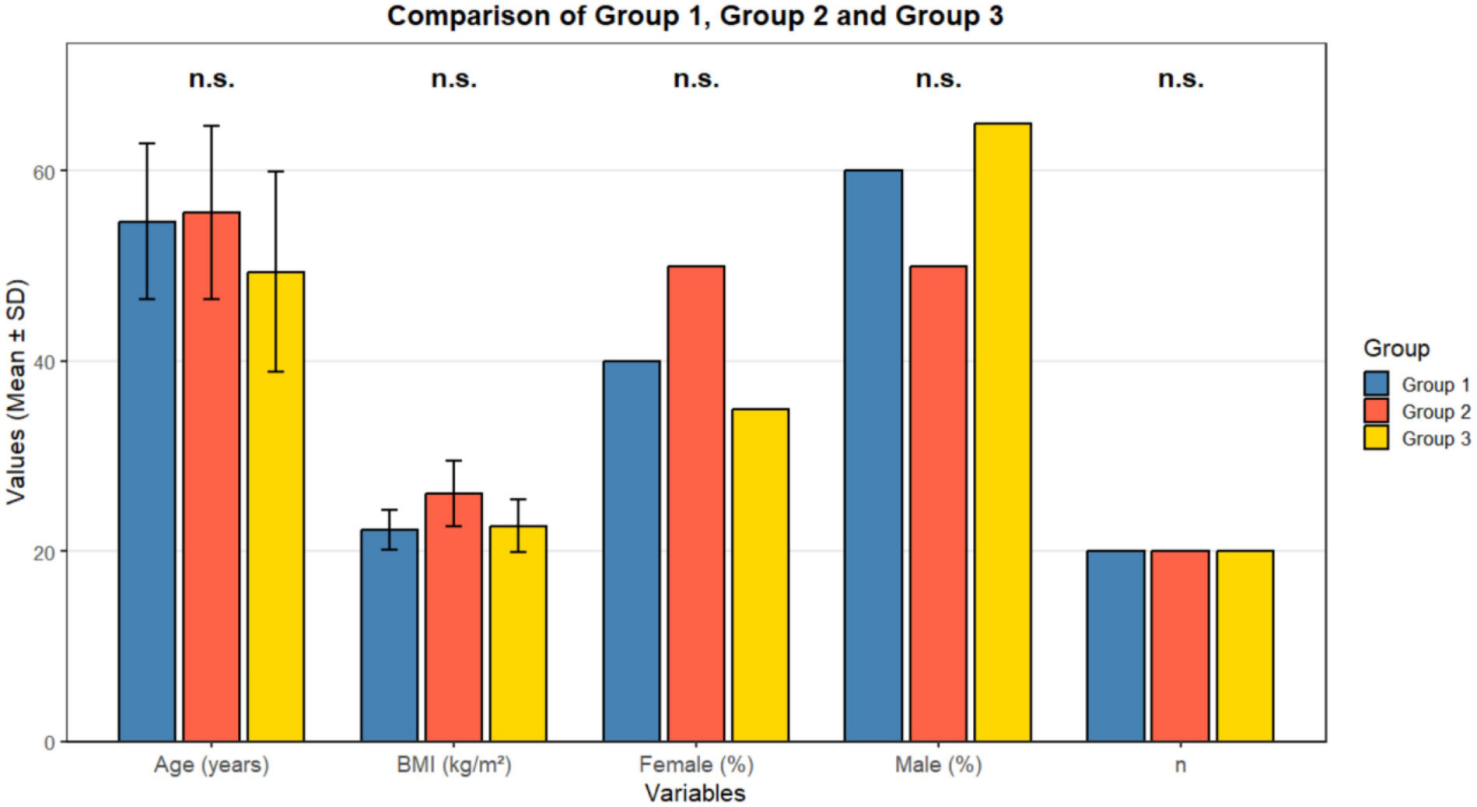
Key parameters of three groups.

**Figure 2:**
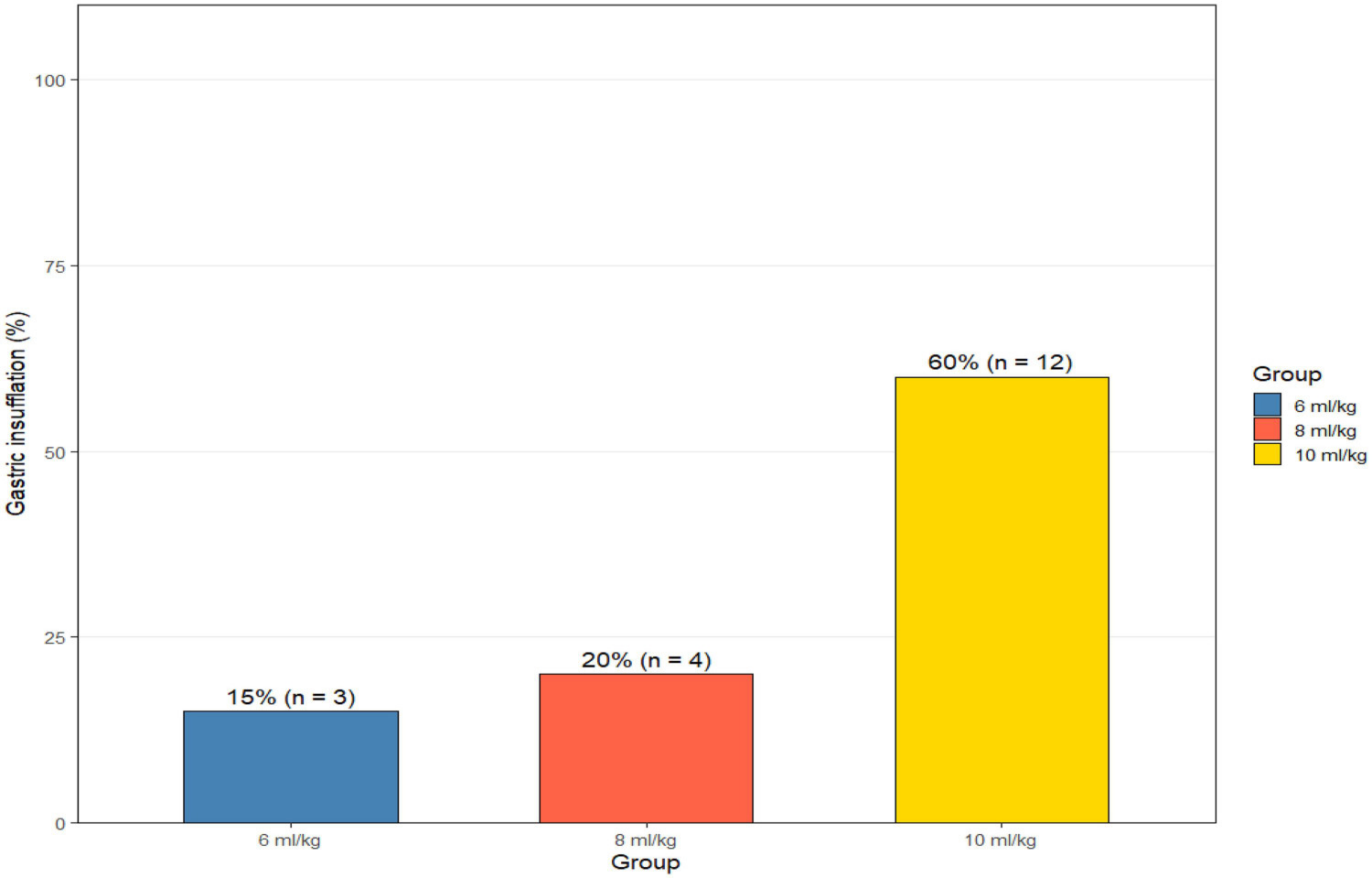
Gastric insufflation data from three group.

Patient demographics were comparable among the three study groups (Table 1). The mean age ranged from 49.4 to 55.6 years, with no significant difference in age distribution (*p* = 0.64). The gender distribution was balanced across groups (*p* = 0.52), and BMI values were also similar (*p* = 0.15). Each group comprised 20 participants: Group 1 had an average age of 54.7 years and BMI of 22.3 kg/m^2^, Group 2 had an average age of 55.6 years and BMI of 26.1 kg/m^2^, and Group 3 had an average age of 49.4 years and BMI of 22.7 kg/m^2^. These data confirm the absence of selection bias among the groups. Figure 1 illustrates the distribution of these baseline characteristics, emphasizing the demographic comparability that strengthens the validity of intergroup comparisons. The incidence of gastric insufflation differed significantly among the groups (*p* < 0.001, Table 2). Group 1 (6 mL/kg) recorded gastric insufflation in 3 patients (15%), Group 2 (8 mL/kg) in 4 patients (20%), and Group 3 (10 mL/kg) in 12 patients (60%). The difference in incidence between the high-volume group (Group 3) and both lower-volume groups was statistically significant (Group 1 vs. Group 3, *p* = 0.0079; Group 2 vs. Group 3, *p* = 0.0225). These results indicate a strong positive relationship between tidal volume and gastric insufflation frequency. The odds of developing gastric insufflation in the 10 mL/kg group were approximately fourfold higher compared to the 6 mL/kg group (aOR: 3.2, 95% CI: 1.8–5.6). Figure 2 visually demonstrates this trend, showing a marked rise in gastric insufflation rate with increasing tidal volume. This confirms that higher ventilation volumes substantially elevate the risk of gastric insufflation even under otherwise controlled ventilation conditions.

**Table 1:** Baseline characteristics.

Variable	Group 1 (6 mL/kg)	Group 2 (8 mL/kg)	Group 3 (10 mL/kg)	*p*-value
Patients (n)	20	20	20	—
Age (years)	54.7 ± 8.2	55.6 ± 9.1	49.4 ± 10.5	0.64
Female (%)	40	50	35	0.52
BMI (kg/m^2^)	22.3 ± 2.1	26.1 ± 3.4	22.7 ± 2.8	0.15

**Table 2:** Primary outcomes.

Group	Gastric Insufflation (%)	Adjusted OR (95% CI)	*p*-value
6 mL/kg	15	Reference	—
8 mL/kg	20	0.32 (0.14–0.71)	0.005
10 mL/kg	60	3.2 (1.8–5.6)	<0.001

Multivariate analysis confirmed that tidal volume group independently predicted gastric insufflation after adjusting for BMI (*β* = 0.41, 95% CI: 0.28–0.53, *p* < 0.001), age (*β* = 0.02, 95% CI: −0.01–0.05, *p* = 0.18), and gender (*β* = 0.15, 95% CI: −0.07–0.37, *p* = 0.17). The 8 mL/kg group showed significantly lower adjusted odds of insufflation compared to 10 mL/kg (aOR: 0.32, 95% CI: 0.14–0.71), while BMI demonstrated a modest but non-significant effect (aOR: 1.08 per kg/m^2^, 95% CI: 0.98–1.19). The multivariable model explained 42% of variance in gastric insufflation (Nagelkerke *R*^2^ = 0.42). Tidal volume remained strongly predictive (10 vs. 8 mL/kg: aOR: 3.2, 95% CI: 1.8–5.6) after adjustment. *post-hoc* comparisons showed all inter-group differences remained significant (*p* < 0.05) after Tukey correction.

Table 3 presents changes in antral cross-sectional area before and after facemask ventilation. A statistically significant increase in antral area was observed in Group 3 (504.1 ± 109.8 mm^2^ after ventilation vs. 420.1 ± 47.1 mm^2^ before, *p* = 0.001). Groups 1 and 2 showed smaller, non-significant changes (*p* > 0.05). Subgroup analysis revealed that within Group 3, patients with gastric insufflation (GI+) exhibited substantially larger post-ventilation antral areas than those without insufflation (GI−) (*p* < 0.001). These findings confirm that high tidal volume ventilation is associated with measurable gastric distension, objectively verified by ultrasound imaging.

**Table 3:** Gastric antral cross-sectional area before and after facemask ventilation in the three tidal volume groups and GI subgroups (mm^2^).

Group	*n*	Antral area before facemask ventilation, mm^2^	Antral area after facemask ventilation, mm^2^	*P*-value
Group 1	20	388.6 ± 54.9	419.7 ± 49.1	0.139
GI+	4	390.6 ± 12.9	411.7 ± 53.1	0.721
GI−	16	389.5 ± 60.1	419.5 ± 50.2	0.169
Group 2	20	399.9 ± 42.9	426.3 ± 44.1	0.129
GI+	3	379.4 ± 24.8	440.4 ± 39.9	0.269
GI−	14	404.7 ± 45.9	419.8 ± 44.8	0.311
Group 3	20	420.1 ± 47.1	**504.1 ± 109.8**	**0.001**
GI+	5	430.1 ± 45.2	**569.5 ± 98.2**	**<0.001**
GI−	15	403.8 ± 49.1	415.7 ± 49.9	0.439

GI, gastric insufflation; GI+, with gastric insufflation; GI−, without gastric insufflation; mm^2^, square millimetres.

Bold values indicate a statistically significant difference before vs. after facemask ventilation within the same group/subgroup (*P* < 0.05).

Table 4 summarizes respiratory parameters (PetCO_2_, ETO_2_, and PIP) recorded during facemask ventilation at 30, 60, 90, and 120 s. Group 3 displayed the highest PIP values and lowest PetCO_2_ levels across time intervals (*p* < 0.001), consistent with greater airway pressures induced by high tidal volume ventilation. Group 2 maintained stable PIP and PetCO_2_ levels throughout, suggesting optimal gas exchange without excessive airway pressure. ETO_2_ levels rose progressively across all groups, reflecting adequate preoxygenation, but differences between groups were not statistically significant (*p* > 0.05). To improve clarity, redundant time-point details were omitted, and key intergroup differences are emphasized.

**Table 4:** End-tidal carbon dioxide (PetCO_2_, mmHg) during facemask ventilation at 30, 60, 90, and 120 s in the three tidal volume groups.

Variables	Group	*n*	30 s PetCO_2_ (mmHg)	60 s PetCO_2_ (mmHg)	90 s PetCO_2_ (mmHg)	120 s PetCO_2_ (mmHg)
PetCO_2_ (mmHg)	Group 1	20	37.4 ± 1.2*	39.1 ± 1.3^*^^,^^§^	39.8 ± 1.5^*^^,^^§^^,^^**^	43.2 ± 1.7^*^^,^^§^^,^^**^^,^^†^
	Group 2	20	34.9 ± 1.3	34.9 ± 1.5	34.9 ± 1.3	34.9 ± 1.5
	Group 3	20	34.8 ± 1.2^‡^	33.3 ± 1.7^‡^^,^^§^	29.9 ± 1.7^‡^^,^^§^^,^^**^	30.1 ± 1.7^‡^^,^^§^^,^^**^^,^^†^
	*p*-value	<0.001	<0.001	<0.001	<0.001	

* *p* < 0.05 vs. Group 2 at the same time point.

** *p* < 0.01 vs. Group 2 at the same time point.

† *p* < 0.05 vs. Group 3 at the same time point.

‡ *p* < 0.05 vs. 60 s within the same group.

§ *p* < 0.05 vs. 30 s within the same group.

Abbreviations: PetCO2, end-tidal carbon dioxide; s, seconds.

In summary, the results demonstrate that tidal volume is the principal determinant of gastric insufflation during facemask ventilation. Moderate tidal volumes (8 mL/kg) provided sufficient oxygenation and minimized gastric insufflation risk, while high volumes (10 mL/kg) markedly increased gastric distension without additional oxygenation benefit.

## 4 Discussion

The primary objective of this study was to evaluate the impact of different ventilation volumes on gastric insufflation in patients undergoing laparoscopic cholecystectomy. Our findings indicated significant differences among the groups, particularly highlighting that moderate ventilation volumes (8 mL/kg) are most effective in minimizing gastric insufflation while maintaining stable respiratory parameters.

The physiological link between tidal volume and gastric insufflation primarily involves airway and esophageal pressures. When tidal volumes increase, peak inspiratory pressure (PIP) and airway pressure rise. If these pressures exceed the lower esophageal sphincter opening pressure (approximately 15–20 cmH_2_O), gas may enter the stomach rather than the lungs. This leads to gastric distension, impairs diaphragmatic excursion, and increases the risk of regurgitation and aspiration. Conversely, moderate tidal volumes maintain effective alveolar ventilation while keeping airway pressures below this threshold, thereby minimizing gastric insufflation.

Our findings align with but extend prior work in critical ways. While Tianliang et al. (9) similarly identified 8 mL/kg as optimal, their study lacked ultrasound quantification of gastric distension. Conversely, Bouvet et al. (15) used ultrasonography but focused only on pressure-controlled ventilation. By combining these approaches, our study provides quantitative evidence of the volume-dependent effect on gastric insufflation, demonstrating a 68% lower insufflation odds at 8 mL/kg (aOR: 0.32; 95% CI: 0.14–0.71) compared with 10 mL/kg.

This reduction exceeds the 50% improvement reported in pediatric studies using pressure-limited strategies (14), suggesting that adult laparoscopy may require distinct thresholds. Our ultrasound-based antral area measurements provide an objective validation of this relationship and extend the mechanistic understanding of tidal volume-induced gastric insufflation.

This physiological relationship is further supported by prior studies indicating that airway pressures above 18–20 cmH_2_O trigger gastric insufflation during facemask ventilation (15). Hence, controlling tidal volume and maintaining airway pressures below this critical limit is crucial.

The clinical implications of our findings are twofold. First, the significantly lower incidence of gastric insufflation in Group 2 (8 mL/kg) suggests an optimal tidal volume range that balances adequate oxygenation (as evidenced by stable ETO_2_ levels) with minimal gastric distension (*p* < 0.001 vs. Group 3). This “sweet spot” aligns with emerging evidence supporting intermediate-volume ventilation in laparoscopic procedures (19). Second, our ultrasound-based quantification of antral area changes provides an objective metric for future studies, addressing the limitations of qualitative assessments in prior research (14, 15). These findings strengthen the case for personalized ventilation strategies based on patient physiology rather than fixed thresholds (19), particularly given the known variability in diaphragmatic compliance during pneumoperitoneum (4). In summary, higher tidal volumes increase intrathoracic and gastric pressures, potentially exceeding esophageal sphincter resistance, whereas moderate tidal volumes provide sufficient alveolar ventilation without triggering gastric insufflation.

These findings have important practical implications for perioperative ventilation management. In routine laparoscopic surgery, adopting a moderate tidal volume of approximately 8 mL/kg during facemask ventilation may reduce gastric insufflation without compromising oxygenation, providing a balanced approach that protects both pulmonary and gastrointestinal systems. In clinical protocols, this could help standardize pre-intubation ventilation settings and reduce reliance on higher volumes that offer no oxygenation benefit but substantially increase gastric distension risk. For obese patients, who have reduced functional residual capacity and higher airway pressures, the relationship between tidal volume and gastric insufflation may be even more pronounced. Altered respiratory mechanics and elevated intra-abdominal pressure in obesity suggest that the optimal tidal volume threshold may differ, underscoring the need for individualized strategies and further dedicated studies in this population.

In Group 1, which received low ventilation volumes, the antral area measurements post-ventilation showed a modest increase, indicating some degree of gastric insufflation. This is consistent with previous studies, such as Lee et al. (14), which demonstrated that lower ventilation volumes could still result in gastric insufflation due to insufficient airway pressures during facemask ventilation. Group 2, receiving moderate ventilation volumes, showed the least gastric insufflation. This group's outcomes align with the findings of Tianliang et al. (9), who reported that a ventilation volume of 8 mL/kg significantly reduced the incidence of gastric insufflation while ensuring effective lung ventilation. Additionally, the stable PetCO_2_ and ETO_2_ levels in this group suggest that moderate ventilation volumes strike an optimal balance between adequate preoxygenation and minimal risk of gastric insufflation. Conversely, Group 3, with high ventilation volumes, experienced the most significant gastric insufflation. The substantial increase in antral area measurements and elevated PetCO2 levels underscore the respiratory challenges associated with higher ventilation volumes. Similar findings were reported by Aydin et al. (25), who compared pressure-controlled ventilation (PCV) and volume-controlled ventilation (VCV) in laparoscopic cholecystectomy, highlighting that higher tidal volumes can exacerbate gastric insufflation and respiratory complications.

Our findings highlight a critical balance in ventilation management: while higher tidal volumes (10 mL/kg) increased gastric insufflation risk by 3-fold compared to 8 mL/kg (aOR: 0.32, 95% CI: 0.14–0.71), they did not significantly improve oxygenation parameters (ETO_2_ 89.8% vs. 90.1%, *p* = 0.34). This aligns with the “lung-protective” vs. “gastric-protective” ventilation paradigm described by Michard et al. (26), where marginal oxygenation gains must be weighed against aspiration risks. Notably, our 8 mL/kg group achieved satisfactory oxygenation (SpO_2_ > 98% in all cases) while keeping Grade 3–4 distension rates at 5%, compared to 25% in the 10 mL/kg group. This suggests moderate volumes may offer the optimal compromise, particularly when combined with PEEP (5–7 cmH_2_O) to maintain alveolar recruitment without elevating gastric pressures (14).

Our study's results underscore the critical need to optimize ventilation strategies during laparoscopic procedures. Effective ventilation not only improves surgical outcomes but also minimizes complications such as regurgitation and aspiration, which are often associated with excessive gastric insufflation. This aligns with the findings of Koivusalo et al. (27), who emphasized the importance of tailored ventilation strategies to mitigate respiratory and gastric complications during laparoscopic surgeries.

The implications of our study are significant for perioperative care in laparoscopic cholecystectomy. The use of moderate ventilation volumes could lead to fewer intraoperative complications, enhancing patient safety and recovery. This is particularly important given the increasing prevalence of laparoscopic procedures and the need for effective anesthesia management to ensure optimal outcomes. The results are in line with the conclusions drawn by Park et al. (7), who found that protective lung ventilation strategies during laparoscopic surgeries can reduce postoperative pulmonary complications and improve patient recovery.

Our findings both confirm and advance prior research in this field, demonstrating that moderate tidal volumes (8 mL/kg) optimally minimize gastric insufflation while maintaining oxygenation a result consistent with Tianliang et al.'s observations (9), though our study significantly strengthens these conclusions through quantitative ultrasound measurements of antral area changes absent in their work. Where Bouvet et al. (15) established ultrasonography's value for detecting insufflation, they focused exclusively on pressure-controlled ventilation, leaving open the question of volume-specific effects that we directly address here with our multivariate-adjusted results (aOR: 0.32, 95% CI: 0.14–0.71 for 8 vs. 10 mL/kg). The magnitude of risk reduction we observed surpasses the 50% improvement reported in pediatric pressure-limited studies (14), suggesting adult laparoscopic procedures may require distinct ventilation thresholds, particularly given the added complexity of pneumoperitoneum effects on respiratory mechanics (4).Together, these findings offer a mechanistic and quantitative rationale for adopting moderate tidal volumes (8 mL/kg) as the safest and most physiologically balanced approach during anesthesia induction.

As a retrospective study, group assignments were based on clinical records rather than randomization, which may introduce selection bias. However, ventilation volumes were standardized per hospital protocol, minimizing variability. While we minimized selection bias through strict inclusion criteria, residual confounding may exist from unmeasured variables like preoperative gastric volume. Our single-center design and specialized ultrasound protocol may limit generalizability. As a retrospective observational study, our design limits the ability to draw definitive causal conclusions between tidal volume and gastric insufflation. Group assignments were based on clinical records and the tidal volumes set according to institutional protocol rather than random allocation, which may introduce selection bias and confounding. To minimise selection bias, we analysed routine clinical data from all consecutive patients who met prespecified inclusion and exclusion criteria during the study period, and ventilation management followed a standardized departmental protocol rather than individual clinician discretion. We also adjusted for key covariates (age, sex, BMI) using multivariable logistic regression. Nevertheless, residual confounding and selection bias from unmeasured factors (such as preoperative gastric volume or subtle differences in anaesthetic management) cannot be excluded. A key strength of this study is the use of routine clinical data and clearly defined inclusion and exclusion criteria, which helped reduce variability in patient characteristics and perioperative management. Another strength is the objective, quantitative assessment of gastric insufflation using standardized gastric ultrasound measurements of antral area, thereby minimizing interpersonal bias. Appropriate statistical methods, including multivariable logistic regression, were applied to account for potential confounding. However, the study has several limitations that should be addressed in future research. First and most critically, the modest sample size (*n* = 60) and single-center design may substantially limit the generalizability of our findings, particularly for high-risk subgroups like morbidly obese (BMI ≥35) or elderly (>80 years) patients. In addition, subgroup analyses, especially comparisons between GI+ and GI− patients are likely underpowered due to the limited sample size, and these results should be interpreted cautiously. While these constraints are common in preliminary laparoscopic ventilation studies they underscore the need for multi-center validation. As well as we demonstrated high inter-rater reliability, subtle variations in probe positioning could still influence antral measurements despite standardized protocols. Moreover, although repeated measurements by the same operator were averaged to minimise intra-observer variability, this was not evaluated with a separate statistical analysis and some residual measurement error cannot be excluded. While *post-hoc* analysis confirmed adequate power (82%) to detect large effect sizes (Cohen's *h* ≥ 0.8) in gastric insufflation incidence, our sample may be underpowered for smaller (but clinically meaningful) differences. Future multicenter studies should conduct *a priori* power calculations based on these preliminary effect estimates. Second, while ultrasonography provided objective measurements of gastric insufflation, other techniques such as transesophageal echocardiography or gastric tonometry might offer additional physiological insights. Third, while our 4-point distension scale showed good inter-rater reliability (*κ* = 0.79), future studies could benefit from standardized tools combining quantitative ultrasound measurements with surgical field assessments to further validate this grading system. Fourth, while our study included patients across a broad age range (29–84 years) and BMI spectrum (mean 22.3–26.1 kg/m^2^), the sample size precluded subgroup analyses by BMI category or age decade. Future studies should explicitly evaluate whether the optimal tidal volume (8 mL/kg) remains consistent in underweight (BMI <18.5) or obese (BMI ≥30) patients, given known differences in respiratory mechanics (28, 29). Fifth, while our multivariate analysis adjusted for key demographic variables, unmeasured confounders (e.g., preoperative gastric volume) may influence insufflation risk. Future studies should incorporate more comprehensive covariates, including esophageal pressure monitoring (4). Sixth, while our comparisons highlight consistency with international studies (7, 15, 19), differences in pneumoperitoneum pressures (11–17 mmHg vs. others' 12–15 mmHg) may limit direct extrapolation. Finally, the study did not evaluate long-term postoperative outcomes related to different ventilation strategies, which could provide a more comprehensive understanding of their clinical impact.

Future research should also explore the impact of different intra-abdominal insufflation pressures on gastric insufflation. Previous studies, such as those by Sefr et al. (30), have shown that varying insufflation pressures can significantly affect arterial blood gas changes and acid-base balance during laparoscopic surgery. Understanding the interplay between insufflation pressure and ventilation volume could lead to more refined strategies for managing gastric insufflation. Moreover, long-term studies examining the effects of different ventilation strategies on postoperative outcomes are warranted. This includes investigating the impact on respiratory and gastrointestinal complications, which would provide a more comprehensive understanding of the best practices for ventilation management in laparoscopic cholecystectomy.

## 5 Conclusion

Our study highlights the importance of optimizing ventilation volumes to reduce gastric insufflation during laparoscopic cholecystectomy. Moderate ventilation volumes, as demonstrated by Group 2, appear to be the most effective in balancing adequate preoxygenation with minimal gastric insufflation. These findings provide a foundation for improving anesthesia management and patient safety in laparoscopic procedures. Further research with larger sample sizes and additional clinical settings is needed to confirm these results and refine ventilation strategies for diverse patient populations.

## Data Availability

The raw data supporting the conclusions of this article will be made available by the authors, without undue reservation.
